# The Final Word

**Published:** 1905-07

**Authors:** 


THE FINAL WORD.—An editorial in the June number of the *Texas Medical Gazette*, Fort Worth (which I attributed to Dr. Chase, one of the editors, but which I learn he did not write), makes it appear that I opposed the founding of a journal for the Association, actuated by interested motives. This is most remarkable, because the writer of the editorial was present in the House of Delegates and heard me say, "If, on further investigation,—which I recommend,—it can be shown that it is the wish of the large membership (and they ought to be consulted), and that we are financially able to do it, no one will more earnestly support the measure than I,"—a sentiment loudly applauded. And further,—I distinctly disclaimed the wish either to edit the journal or to have the "Red Back" made the official organ. I now want it understood that I *did not* and *do not* oppose it, but earnestly wish it may succeed and prove acceptable. But I still regard it as a hazardous experiment, which time alone will determine. Don't shed any tears about the "shorn lamb" at which the *Gazette* hints. I can take care of myself, and I'll be doing business at the old stand when the *Gazette* turns up its toes to the daisies,—and fighting for the best interests of the profession as I see it, as I have always done, and for the rights of the rank and file,—the boys from up the creek,—the men behind the *money,*—and they appreciate it. Selah. But if, as in some other States, its editorial pages are to be prostituted to *whipping in subscribers for the Octopus,* and coercing men into membership, it does not deserve to succeed. Are not there many—a great many good, pure and able men outside of the organizations as there are inside, and who *do not want to become members?* Sure. Fully half of my subscribers are not members. Fair play and a consideration for their rights and opinions is what I ask for them, as well as for all members and for myself.

